# Isolated right fallopian tube torsion associated with hydrosalpinx and multiple uterine leiomyomas in a 50-year-old woman: a case report

**DOI:** 10.3389/frph.2026.1904637

**Published:** 2026-09-07

**Authors:** Khaled F. Al-Ali, Zaid Al Ali, Dima M. Alem, Ruba Abdalhadi, Yazan Dibas, Samaa' Milhem, Saadeh Jaber

**Affiliations:** 1 Faculty of Medicine, Al-Quds University, Jerusalem, Palestine; 2 Department of Radiology, Al-Makassed Islamic Charitable Hospital, Jerusalem, Palestine; 3 Department of Obstetrics and Gynecology, Al-Makassed Islamic Charitable Hospital, Jerusalem, Palestine

**Keywords:** acute abdominal pain, acute pelvic pain, hydrosalpinx, IFTT, isolated tubal torsion

## Abstract

**Background:**

Isolated fallopian tube torsion (IFTT) is a rare gynecologic emergency that frequently presents with nonspecific symptoms and may mimic other causes of acute abdominal pain, resulting in diagnostic delay.

**Case presentation:**

A 50-year-old woman presented with a 2-day history of right lower quadrant pain without gastrointestinal, urinary, or gynecologic symptoms and was initially admitted with suspected appendicitis. Pelvic ultrasonography demonstrated right hydrosalpinx with absent Doppler flow, while contrast-enhanced computed tomography revealed a 6 cm × 2.5 cm hydrosalpinx with a possible whirlpool sign, multiple calcified uterine fibroids, and a cervical lesion; the appendix was normal. Based on the clinical and radiologic findings, right fallopian tube torsion was suspected. Owing to the presence of multiple large fibroids and concern for possible sarcomatous change, the patient underwent total abdominal hysterectomy with bilateral salpingo-oophorectomy. Intraoperatively, a gangrenous, torsed right fallopian tube and a bulky, fibroid uterus were identified. Histopathological examination confirmed ischemic necrosis of the right fallopian tube consistent with torsion and demonstrated benign leiomyomata with hyalinization and calcification, without evidence of malignancy.

**Conclusion:**

IFTT should be considered in women presenting with acute unilateral lower abdominal pain. Early recognition and prompt surgical intervention are essential for definitive diagnosis and prevention of adverse outcomes.

## Introduction

Isolated fallopian tube torsion (IFTT) is a rare gynecological emergency, with an estimated incidence of approximately 1 in 1.5 million women (1). It is characterized by torsion of the fallopian tube in the absence of ovarian involvement, resulting in vascular compromise (2). The condition is thought to arise from impaired venous and lymphatic drainage, leading to tubal congestion, edema, and subsequent ischemia. Ovarian involvement is usually absent because tubal perfusion may be compromised while ovarian blood flow remains preserved through the distinct vascular supply of the adnexa (3). Patients typically present with acute lower abdominal pain, with or without nausea, vomiting, or fever, often mimicking other gynecologic and nongynecologic conditions and contributing to delayed diagnosis (2).

The etiology of isolated fallopian tube torsion (IFTT) remains incompletely understood, although several predisposing factors have been described (4). These factors can be broadly classified into intrinsic tubal abnormalities and extrinsic pelvic factors. Intrinsic factors include congenital tubal elongation or excessive mobility, as well as acquired conditions such as hydrosalpinx, hematosalpinx, pyosalpinx, endometriosis, neoplasms, and abnormal tubal motility. Extrinsic factors include prior pelvic surgery, adhesions, pelvic trauma, pregnancy, pelvic congestion, and adjacent adnexal or pelvic masses. Among these, hydrosalpinx is a recognized risk factor due to tubal distension and increased mobility, which may predispose the tube to torsion (2, 5).

Recent evidence suggests that isolated fallopian tube torsion comprises distinct anatomical and etiological subtypes that may differ in their underlying pathogenesis. Three laparoscopic subtypes have been described: Type 1, organoaxial torsion without an associated lesion; Type 2, organoaxial torsion associated with a leading mass, most commonly a paratubal cyst; and Type 3, mesenteroaxial torsion around the tube's short axis. Hydrosalpinx is reported more frequently in Types 1 and 3, whereas paratubal cysts are typically associated with Type 2 (6).

Despite advances in imaging, preoperative diagnosis remains challenging because clinical manifestations are nonspecific and radiologic findings are often inconclusive (7). Consequently, IFTT is frequently diagnosed only at the time of surgical exploration (2). No imaging modality is diagnostic in isolation, and a high index of suspicion remains essential when evaluating patients with acute pelvic pain. Laparoscopy continues to represent the diagnostic and therapeutic standard, providing definitive assessment of tubal viability and facilitating timely management (8).

## Case presentation

### Patient profile

A 50-year-old single woman, who reported that she had never engaged in sexual intercourse, presented to the emergency department with a 2-day history of right lower abdominal pain. The pain was not associated with gastrointestinal, urinary, or gynecologic symptoms. She was initially admitted under the surgical team for evaluation of suspected appendicitis. Her menstrual history was unremarkable, with menarche at 12 years of age, regular 21-day cycles lasting 5 days, and no history of dysmenorrhea. She had no significant medical or surgical history, was not taking any regular medications, and reported no known drug allergies or family history of gastrointestinal, gynecologic, or breast malignancy.

On presentation, she was hemodynamically stable, with a blood pressure of 140/87 mmHg, a pulse rate of 90 beats/min, and a temperature of 36.3 °C. Her body mass index was 23 kg/m^2^. Abdominal examination, performed after administration of analgesia, revealed a soft, non-tender abdomen without signs of peritoneal irritation. Gynecologic examination demonstrated a bulky, irregular, non-tender uterus, with no adnexal mass or other remarkable findings.

### Diagnostic assessment

**Figure 1:**
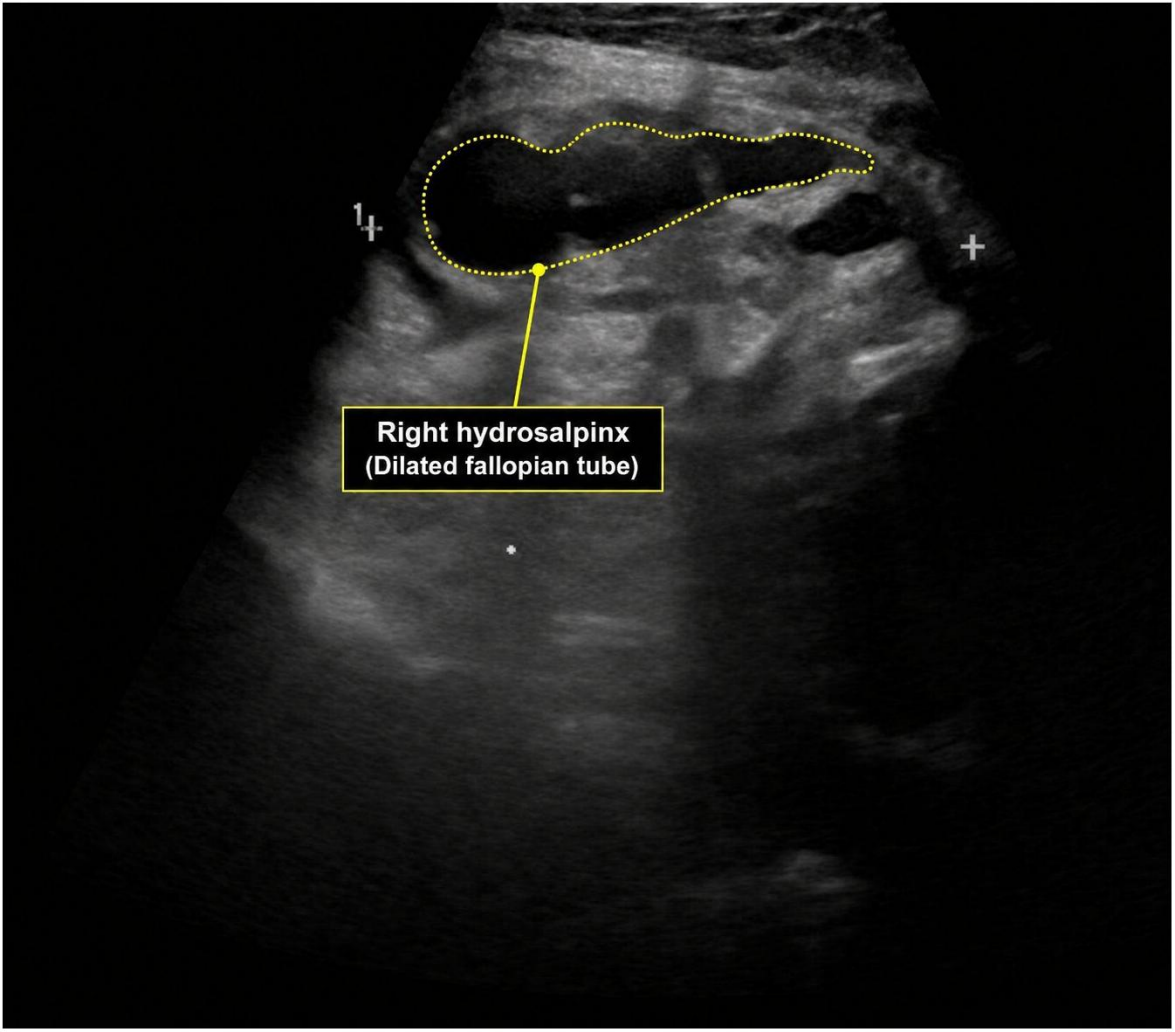
Transabdominal pelvic ultrasonography demonstrating a right hydrosalpinx with dilatation of the fallopian tube and an anechoic cystic tubular structure within the right adnexal region.

Initial laboratory investigations were unremarkable, with a hemoglobin level of 13.3 g/dL, white blood cell count of 6.6 × 10^9^/L, platelet count of 295 × 10^9^/L, and mildly elevated C-reactive protein of 13.1 mg/L. Renal function was within normal limits, and serum β-hCG was negative. Pelvic ultrasonography demonstrated a right hydrosalpinx, visualized as a dilated tubular cystic structure within the right adnexa, with minimal free fluid in the pouch of Douglas (Figure 1). Doppler evaluation revealed absent vascular flow within the affected tube, raising suspicion for isolated right fallopian tube torsion. The uterus was enlarged, containing a posterior intramural fibroid measuring 8 cm × 7 cm with internal calcification.

**Figure 2:**
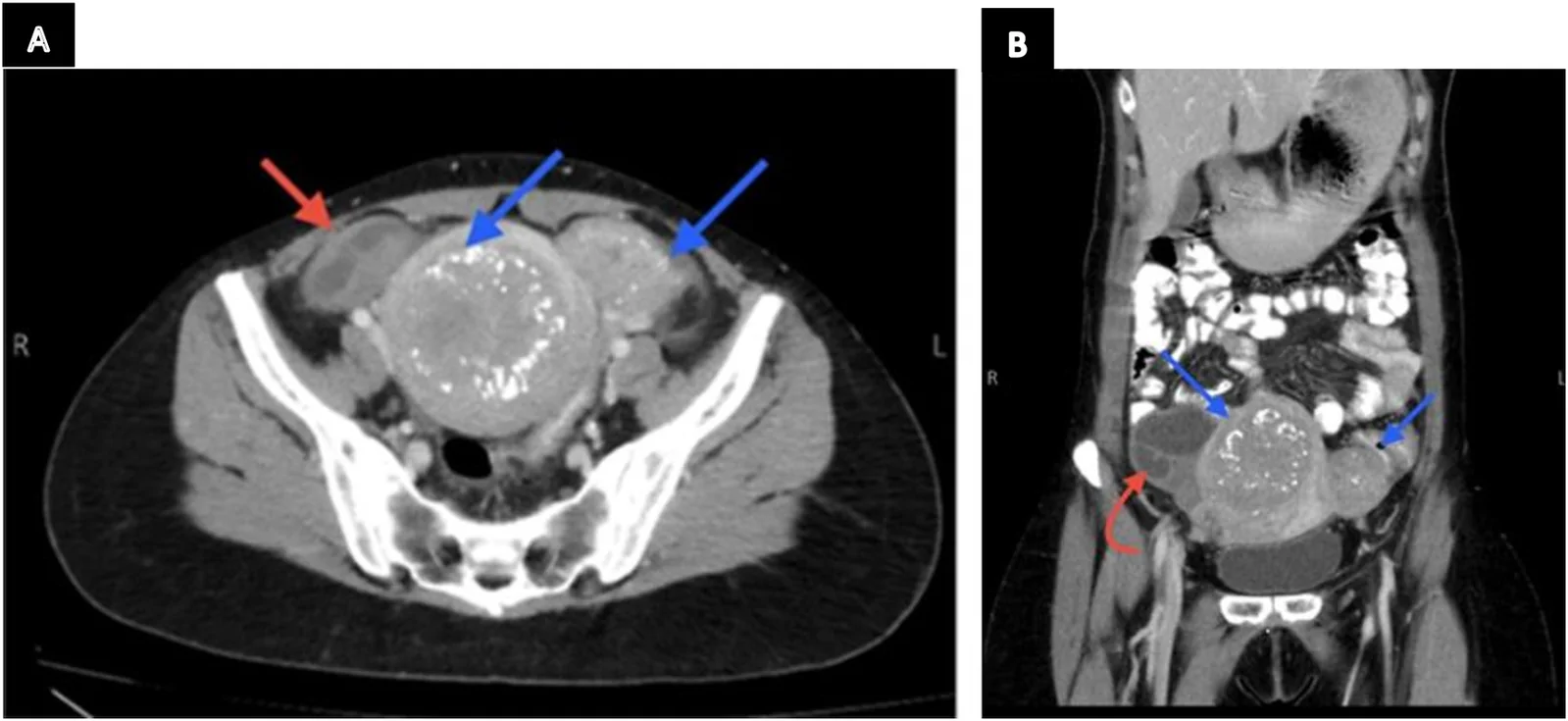
Contrast-enhanced computed tomography of the abdomen and pelvis demonstrating a dilated cystic tubular structure in the right adnexal/right iliac fossa region, consistent with hydrosalpinx (red arrow), together with calcified uterine fibroids (blue arrows). **(A)** Axial view. **(B)** Coronal view.

To further characterize these findings and exclude alternative pathology, a subsequent contrast-enhanced computed tomography (CE-CT) scan of the abdomen and pelvis was performed. This demonstrated a 6 cm × 2.5 cm thick-walled tubular cystic lesion adjacent to the right ovary, consistent with hydrosalpinx, with a possible whirlpool sign suggestive of torsion. Additional findings included an 8 cm calcified intramural fibroid, a second 4 cm calcified pelvic lesion likely representing an additional fibroid, and a 2.4 cm cervical lesion (Figure 2), while the appendix appeared normal. Given the complex pelvic findings and radiologic concern for possible malignant transformation, serum CA 19-9 (14.3 U/mL) and CA-125 (29.5 U/mL) were subsequently assessed and were within normal limits.

### Surgical intervention

A total abdominal hysterectomy with bilateral salpingo-oophorectomy was performed due to clinical and radiologic suspicion of right fallopian tube torsion with hydrosalpinx, multiple uterine fibroids, and concern for possible malignant transformation. Following entry into the abdominal cavity, systematic pelvic inspection revealed an enlarged fibroid uterus, a gangrenous right fallopian tube twisted around its axis, a normal left fallopian tube, atrophic ovaries, and a cervical mass. The uterus was enlarged by a 7 cm × 7 cm fundal intramural fibroid and a 3 cm × 3 cm anterior subserosal fibroid. The right fallopian tube torsion was confirmed intraoperatively, with no adhesions or other apparent extrinsic causes identified. The surgical specimen, including the uterus, cervix, bilateral adnexa, and fallopian tubes, was submitted for histopathological examination, which confirmed benign leiomyomata with hyalinization and calcification without evidence of malignancy. The procedure was completed without complications, with an estimated blood loss of 200 mL.

### Gross specimen and histopathological findings

**Figure 3:**
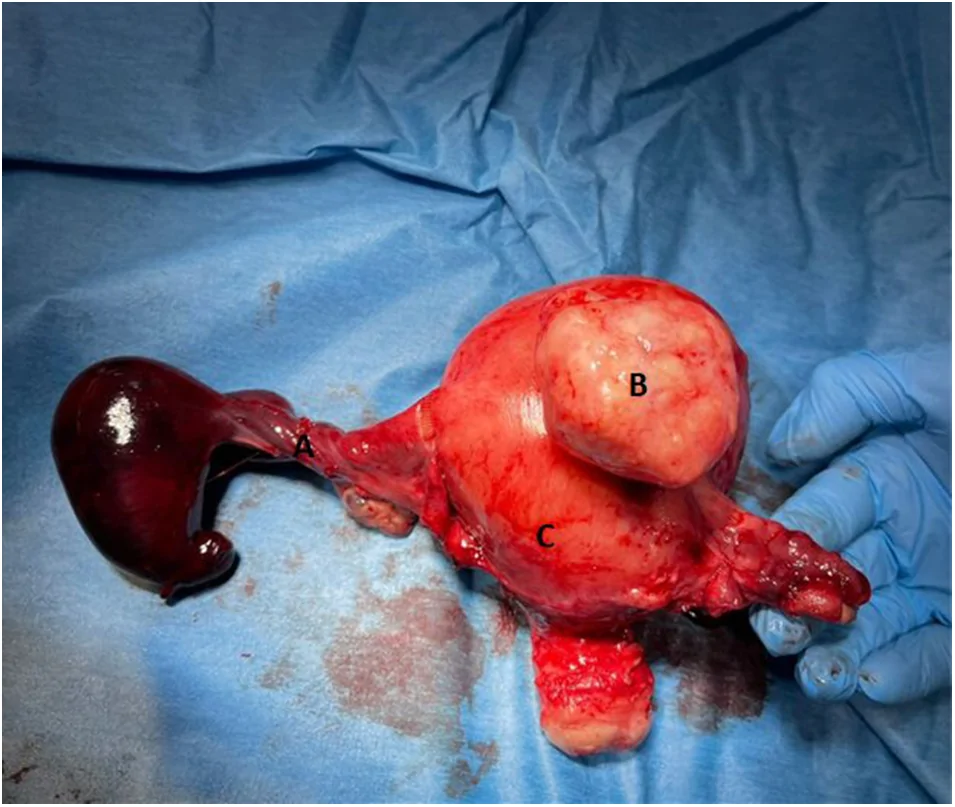
Gross pathological specimen following total abdominal hysterectomy with bilateral salpingo-oophorectomy, showing an enlarged uterus (label **C**) containing multiple intramural and subserosal leiomyomas (label **B**). The right fallopian tube is enlarged, gangrenous, and demonstrates features consistent with torsion (label **A**).

Gross examination of the hysterectomy specimen revealed an enlarged uterus measuring 14 cm × 7 cm × 7 cm with multiple intramural and subserosal leiomyomas, the largest measuring 7 cm × 6 cm × 5 cm. An endometrial polyp measuring 3 cm × 1 cm was also identified. The right fallopian tube was enlarged and gangrenous, with gross features indicative of torsion, while both ovaries and the left fallopian tube were unremarkable (Figure 3).

Microscopic examination showed benign leiomyomata with hyalinization and calcification, without significant atypia, increased mitotic activity, or tumor cell necrosis. Additional findings included focal chronic cervicitis, an endometrial polyp with proliferative changes, and a subserosal leiomyoma associated with adenomyosis. The right fallopian tube demonstrated ischemic necrosis consistent with torsion. No evidence of malignancy was identified.

### Post-operative management

The postoperative course was uneventful. On the first postoperative day, the patient remained hemodynamically stable, pain-free, and without dizziness, palpitations, or shortness of breath. Abdominal examination was unremarkable, the surgical dressing was clean and dry, and no vaginal bleeding was observed. Postoperative ultrasonography demonstrated no hematoma or intra-abdominal collection. However, hemoglobin level decreased from 13.3 g/dL preoperatively to 9.7 g/dL, subsequently improving to 10.0 g/dL, and oral iron supplementation was initiated. By postoperative day two, she remained clinically stable and had resumed bowel function.

### Follow-up and outcomes

The patient remained hemodynamically stable, tolerated oral intake, and recovered without complications. She was reviewed during follow-up, where she reported complete resolution of her abdominal pain. Histopathological findings were discussed with the patient, and no further intervention was required. A brief overview of the patient's clinical characteristics, investigations, and outcomes is in (Table 1).

**Table 1:** Summary of clinical characteristics, investigations, and health outcome of IFTT patient.

Feature	Case
Age/Sex	50-year-old woman
Presentation	Right lower quadrant pain for 2 days without gastrointestinal, urinary, or gynecologic symptoms.
Initial Assessment	Admitted with suspected appendicitis.
Key Imaging Findings	Ultrasound demonstrated right hydrosalpinx with absent Doppler flow. CT showed a 6 cm × 2.5 cm hydrosalpinx with a possible whirlpool sign, multiple uterine fibroids, and a normal appendix.
Laboratory Findings	Hb 13.3 g/dL, WBC 6.6 G/L, CRP 13.1 mg/L, β-hCG negative, CA 125 and CA 19-9 within normal limits.
Final Diagnosis	Isolated right fallopian tube torsion with ischemic necrosis and hydrosalpinx, associated with multiple uterine leiomyomas.
Treatment	Total abdominal hysterectomy with bilateral salpingo-oophorectomy. Intraoperatively, a gangrenous torsed right fallopian tube and multiple uterine fibroids were identified.
Outcome	Uneventful recovery and discharge on postoperative day 2. Histopathology confirmed ischemic necrosis of the right fallopian tube consistent with torsion and showed no evidence of malignancy.

## Patient perspective

The patient experienced significant anxiety after the diagnostic evaluation revealed a pelvic pathology requiring surgical intervention and raised the possibility of an underlying malignancy. Following counseling regarding the findings, treatment options, and rationale for surgery, she understood the proposed management plan and agreed to proceed. At follow-up, she reported complete resolution of her abdominal pain and expressed reassurance after histopathological examination confirmed a benign diagnosis without evidence of malignancy.

## Discussion

Isolated fallopian tube torsion (IFTT) is a rare gynecologic emergency and an uncommon cause of acute lower abdominal pain (2). This report describes a 50-year-old woman with an isolated right fallopian tube torsion, most likely secondary to hydrosalpinx. This case illustrates the diagnostic and therapeutic challenges often encountered in the management of this condition.

IFTT is a rare gynecologic emergency, with an estimated incidence of approximately 1 in 1.5 million women (3). It is clinically important because delayed diagnosis may result in tubal necrosis and irreversible loss of reproductive potential (1, 9). Although it has been described across all age groups, it occurs most frequently in women younger than 30 years (3). Its clinical presentation is highly variable and often nonspecific, typically consisting of acute unilateral lower abdominal pain with or without nausea, vomiting, fever, vaginal bleeding, or lower urinary tract symptoms (10). However, IFTT may also remain entirely asymptomatic and be identified incidentally during surgery for unrelated gynecologic pathology (11). Consequently, IFTT is frequently misdiagnosed because it closely resembles more common surgical and gynecologic emergencies, including appendicitis, ovarian torsion, ruptured ovarian cysts, and ectopic pregnancy (12). Right-sided IFTT is reported more frequently than left-sided disease, possibly because patients with right-sided lower abdominal pain are more likely to undergo surgical exploration for suspected appendicitis, whereas the sigmoid colon may limit mobility of the left adnexa and reduce the risk of torsion (13).

Other important differential diagnoses include tubo-ovarian abscess, degenerating leiomyoma, and paraovarian cyst torsion. Tubo-ovarian abscess typically presents with fever, leukocytosis, elevated inflammatory markers, and a complex multiloculated adnexal mass on imaging, reflecting an underlying pelvic infection (14). Degenerating leiomyoma usually occurs in women with known uterine fibroids and is characterized by localized pain arising from ischemic degeneration of the fibroid rather than adnexal pathology (15). Paraovarian cyst torsion may be particularly difficult to distinguish from isolated fallopian tube torsion because both can present with acute unilateral pelvic pain and a normal ipsilateral ovary on imaging; definitive differentiation often requires surgical exploration (16).

The pathophysiology of IFTT remains poorly understood, although several intrinsic and extrinsic factors have been proposed in its development (3). Intrinsic factors, including hydrosalpinx, hematosalpinx, tubal elongation, congenital anomalies, and abnormal tubal peristalsis, may increase tubal mobility and predispose the tube to torsion (4). In addition, extrinsic factors such as paraovarian or paratubal cysts, pelvic adhesions, pelvic inflammatory disease, and adjacent inflammatory processes may facilitate torsion by providing a fixed point around which the tube can rotate (2, 4). Previous tubal ligation has also been identified as a potential risk factor, likely through hydrosalpinx formation and postoperative adhesions that further increase susceptibility to tubal twisting (17).

Ultrasonography is the first-line imaging modality for the evaluation of suspected gynecologic causes of acute pelvic pain and may assist in the diagnosis of IFTT, although its diagnostic accuracy remains limited, with correct preoperative diagnoses reported in fewer than 30% of cases (2). The most suggestive sonographic finding is a normal ipsilateral ovary adjacent to a tubular adnexal structure, such as a hydrosalpinx, often accompanied by free pelvic fluid (2, 9). Doppler findings may also be inconclusive, as preserved ovarian vascularity does not exclude tubal torsion; however, absent vascular flow in the affected adnexal region and the whirlpool sign, which reflects twisting of the vascular pedicle, may increase clinical suspicion when present (2). Computed tomography (CT) scan or magnetic resonance imaging (MRI) may further characterize adnexal pathology and exclude alternative causes of acute abdominal pain, particularly when the diagnosis remains uncertain (18).

**Figure 4:**
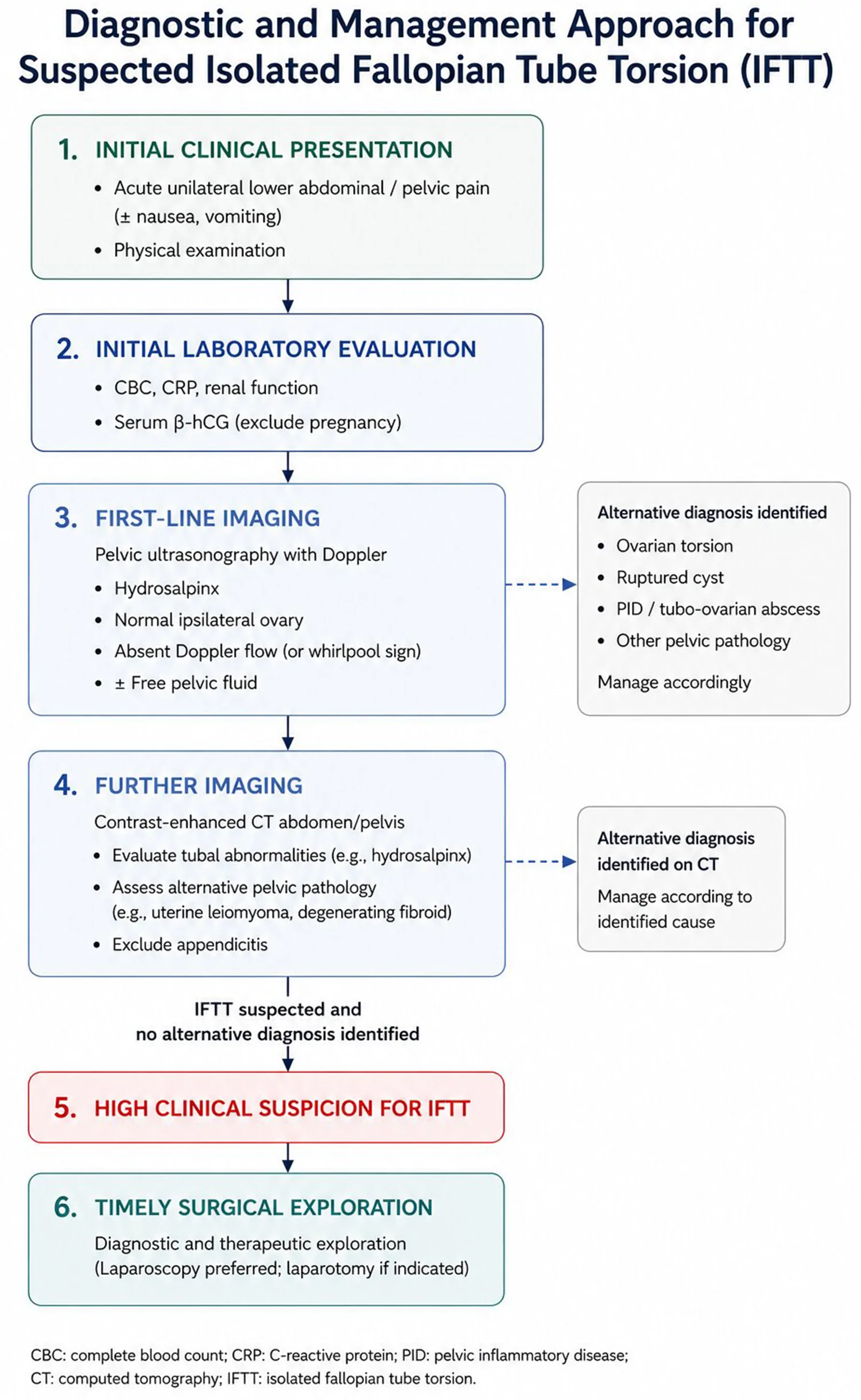
This algorithm outlines the stepwise diagnostic and management approach of suspected isolated fallopian tube torsion (IFTT), from initial assessment to definitive surgical diagnosis and treatment.

The diagnostic evaluation in our case was based on a combination of clinical assessment, laboratory investigations, and complementary imaging modalities. As part of the approach to acute pelvic pain, we performed initial laboratory testing, which included a complete blood count, inflammatory markers, and serum β-hCG (despite the patient's reported lifelong absence of sexual intercourse). Subsequent pelvic ultrasonography demonstrated a right hydrosalpinx with absent Doppler flow, raising suspicion for isolated fallopian tube torsion, while also revealing marked uterine enlargement due to multiple fibroids. To exclude other differentials and further characterize the pelvic findings, contrast-enhanced computed tomography (CE-CT) of the abdomen and pelvis was subsequently performed. It confirmed a normal appendix and demonstrated multiple large uterine fibroids with imaging features concerning for possible malignant transformation. Although no adnexal mass was identified on physical examination or initial ultrasonography, serum CA-125 and CA 19-9 were obtained as part of the preoperative evaluation to assess the possibility of underlying gynecologic malignancy and to assist surgical planning, rather than to establish the diagnosis of isolated fallopian tube torsion. MRI was not pursued because the combined clinical and imaging findings were considered sufficient to justify prompt surgical exploration without delaying definitive management. The diagnostic approach is summarized in Figure 4.

Management of isolated fallopian tube torsion is primarily surgical, with laparoscopy remaining the gold standard for diagnosis and treatment (8). Prompt intervention is essential to prevent irreversible ischemic injury and maximize the potential for fertility preservation. Although salpingectomy has traditionally been the most common surgical approach, increasing emphasis has been placed on conservative management with detorsion and tubal preservation, particularly in pediatric patients and women of reproductive age when tubal viability is preserved. However, concerns regarding recurrence, future fertility outcomes, and the functional recovery of severely compromised tubes continue to influence the choice between salpingectomy and conservative surgery (3, 18). Consequently, management should be individualized, with fertility-preserving strategies favored whenever feasible (5).

In our case, a total abdominal hysterectomy with bilateral salpingo-oophorectomy was performed for suspected isolated fallopian tube torsion in the setting of extensive uterine pathology. The patient presented with acute unilateral lower abdominal pain, and imaging demonstrated a right hydrosalpinx with absent Doppler flow, raising concern for compromised tubal perfusion and supporting prompt surgical exploration. In addition, multiple large uterine lesions with imaging features suspicious for possible malignant transformation favored definitive surgical management over a conservative approach. Intraoperatively, the right fallopian tube was gangrenous and torsed around its axis, confirming isolated fallopian tube torsion. No adhesions or other extrinsic causes were identified, suggesting that hydrosalpinx was the most likely predisposing factor.

Histopathological examination confirmed benign leiomyomata with hyalinization and calcification, with no evidence of malignancy. In addition, the patient experienced a postoperative decline in hemoglobin from 13.3 g/dL to 9.7 g/dL despite an estimated intraoperative blood loss of approximately 200 mL. Because of the retrospective nature of this report, the precise reason could not be determined from the available medical records. Potential contributing factors include perioperative hemodilution from intravenous fluid administration or underestimation of blood loss. Importantly, the patient remained hemodynamically stable throughout the postoperative period, with no clinical or ultrasonographic evidence of ongoing hemorrhage, required no blood transfusion or reoperation, and recovered uneventfully.

This report contributes to the literature by documenting a well-characterized case of isolated fallopian tube torsion from an underrepresented healthcare setting. To the best of our knowledge, it represents the first published case from Palestine, thereby expanding the geographic representation of this rare condition. Although it does not introduce a novel diagnostic or therapeutic strategy, it provides educational value through detailed documentation of the diagnostic evaluation, intraoperative findings, histopathological confirmation, and postoperative outcome. By illustrating the diagnostic challenges encountered in this atypical presentation, this report complements the existing literature and may assist clinicians in the recognition and management of similar cases.

## Conclusion

In conclusion, isolated fallopian tube torsion is a rare cause of acute pelvic pain with nonspecific clinical and imaging features, which makes preoperative diagnosis challenging. Maintaining a high index of suspicion and ensuring timely surgical exploration are essential to reduce diagnostic delay and improve the likelihood of tubal preservation and favorable reproductive outcomes.

## Data Availability

The original contributions presented in the study are included in the article/Supplementary Material, further inquiries can be directed to the corresponding authors.

## References

[1] Babier A Alfaqeh M Makki J Althobaiti R. Acute isolated fallopian tube torsion in a 37-year-old woman: a case report. J Med Case Rep. (2025) 19(1):462. 10.1186/s13256-025-05527-1 41024176 PMC12482216

[2] Pignataro JN Schindler L. Isolated fallopian tube torsion: diagnosis and management of a gynecologic emergency. Cureus. (2023) 15(9):e46260. 10.7759/cureus.46260 37908916 PMC10615115

[3] Gross M Blumstein SL Chow LC. Isolated fallopian tube torsion: a rare twist on a common theme. Am J Roentgenol. (2005) 185(6):1590–2. 10.2214/ajr.04.1646 16304018

[4] Elçi E Sayan S Elçi G Koç G. Isolated fallopian tubal torsion: reproductive age case series. J Obstet Gynaecol Res. (2021) 47(7):2515–20. 10.1111/jog.14810 33913220

[5] Balasubramaniam D Duraisamy K Ezhilmani M Ravi S. Isolated fallopian tube torsion: a rare twist with a diagnostic challenge that may compromise fertility. J Hum Reprod Sci. (2020) 13(2):162. 10.4103/jhrs.jhrs_143_19 32792767 PMC7394095

[6] Takeda A Koike W. Identification of 3 subtypes of isolated fallopian tube torsion in 19 laparoscopically confirmed cases: case series and literature review. J Gynecol Surg. (2023) 39(3):135–41. 10.1089/gyn.2022.0111

[7] Banerjee I Thakur Y Mukherjee G Jadhav J Sahare A. Isolated fallopian tube torsion: a rare entity. Case Rep Obstet Gynecol. (2021) 2021:1–5. 10.1155/2021/3872201 PMC8651354 34888108

[8] Zijun L Yaqin Z Weiwen P. A rare case of isolated fallopian tubal torsion in adolescent girls: a case report and system review of literature. SAGE Open Med Case Rep. (2023) 11:2050313X231215207. 10.1177/2050313x231215207 38047268 PMC10691320

[9] Coutureau J Mandoul C Curros-Doyon F Millet I Taourel P. Recognizing the features of isolated fallopian tube torsion on CT and MRI and interobserver agreement: a cross-sectional study. Eur J Radiol. (2022) 157:110607. 10.1016/j.ejrad.2022.110607 36410090

[10] Ziogas AC Thanasas IK Oikonomou IT Tsiamanta C. Torsion of the left fallopian tube without ovarian involvement in a 47-year-old woman: a case report. Case Rep Womens Health. (2020) 26:e00179. 10.1016/j.crwh.2020.e00179 32082992 PMC7019123

[11] Kale İ Kırmacı C. Isolated fallopian tube torsion—case reports of one symptomatic and one asymptomatic patient. Česká Gynekologie. (2025) 90(4):320–2. 10.48095/cccg2025320 41182873

[12] Meyer R Meller N Mohr-Sasson A Toussia-Cohen S Machtinger R Bart Y. Clinical features of isolated fallopian tube torsion: evidence from a large series. Hum Fertil. (2022) 26(5):971–7. 10.1080/14647273.2022.2034056 35114880

[13] Bashir H Thomas M Abubakar M Satti A. Twisted right fallopian tube with a large fimbrial cyst: a diagnostic challenge in acute pelvic pain. Cureus. (2025) 17(10):e94176. 10.7759/cureus.94176 41210056 PMC12595530

[14] Iraha Y Okada M Iraha R Azama K Yamashiro T Tsubakimoto M. CT And MR imaging of gynecologic emergencies. Radiographics. (2017) 37(5):1569–86. 10.1148/rg.2017160170 28753380

[15] Dawood MT Naik M Bharwani N Sudderuddin SA Rockall AG Stewart VR. Adnexal torsion: review of radiologic appearances. Radiographics. (2021) 41(2):609–24. 10.1148/rg.2021200118 33577417

[16] Liu D Gao Y Wu Y Guo H Zhang K. Clinical features of isolated fallopian tube torsion and tubo-ovarian torsion with ipsilateral paraovarian cysts: a large cohort study of thirteen-year single-centre experience. BMC Womens Health. (2026) 26(1):165. 10.1186/s12905-026-04319-z PMC12998081 41673638

[17] Jajoo S Jajoo S Naval R. Isolated unilateral hydrosalpinx torsion in a post-tubal ligation patient: a case report and review of literature. Cureus. (2024) 16(3):e56351. 10.7759/cureus.56351 38633976 PMC11021850

[18] Aziza B Houas Y Slimani A Jouini R. Isolated fallopian tube torsion in young females: a case series. J Pediatr Surg Case Rep. (2025) 117:102991. 10.1016/j.epsc.2025.102991

